# Foundations and Advancements in Hemodynamic Monitoring: Part II - Advanced Parameters and Tools

**DOI:** 10.4274/TJAR.2025.251926

**Published:** 2025-10-14

**Authors:** Muhammed Enes Aydın, Aslıhan Aykut, Ümit Karadeniz, Emre Sertaç Bingül, Zeliha Aslı Demir, Gamze Talih, Başak Akça, Burhan Dost

**Affiliations:** 1 Atatürk University Faculty of Medicine, Department of Anaesthesiology and Reanimation, Erzurum, Türkiye; 2 University of Health Sciences Türkiye, Ankara Bilkent City Hospital, Clinic of Anaesthesiology and Reanimation, Ankara, Türkiye; 3 İstanbul University, İstanbul Faculty of Medicine, Department of Anaesthesiology and Intensive Care, İstanbul, Türkiye; 4 Erciyes University Faculty of Medicine, Department of Anaesthesiology and Reanimation, Kayseri, Türkiye; 5 Hacettepe University Faculty of Medicine, Department of Anaesthesiology and Reanimation, Ankara, Türkiye; 6 Ondokuz Mayıs University Faculty of Medicine, Department of Anaesthesiology and Reanimation, Samsun, Türkiye

**Keywords:** Anaesthesia monitoring, hemodynamics, intensive care, patient outcomes, perioperative care

## Abstract

Advanced hemodynamic monitoring has revolutionized perioperative medicine and critical care by providing comprehensive insights into cardiovascular physiology and facilitating precise assessment and management of complex parameters such as cardiac output, systemic vascular resistance, fluid responsiveness, and tissue perfusion. These technologies enhance the capacity of clinicians to detect subtle physiological alterations, enabling timely interventions and individualized therapeutic strategies, particularly for critically ill patients and those undergoing major surgical procedures. This two-part review offers a comprehensive analysis of hemodynamic monitoring. Part I examined the fundamental principles of macrohemodynamics and microhemodynamics. Part II focuses on advanced hemodynamic monitoring tools, tracing the evolution of cardiac output measurement techniques from Fick’s oxygen consumption method in 1870 to contemporary innovations, such as pulse contour analysis, bioimpedance/bioreactance, and real-time non-invasive modalities like advanced echocardiography. By examining the underlying principles, devices, invasiveness, clinical applications, advantages, and limitations of various monitoring techniques, this review elucidates the clinical utility of advanced tools in addressing the limitations of standard monitoring and optimizing patient outcomes in modern anaesthesia and critical care practices.

**Main Points**

• Advanced hemodynamic monitoring tools provide precise, real-time assessment of cardiovascular parameters, improving clinical decision-making in perioperative and critical care settings.

• The evolution of cardiac output measurement techniques—from thermodilution and pulse contour analysis to less invasive modalities like echocardiography and bioimpedance—has significantly enhanced the accuracy, practicality, and safety of hemodynamic monitoring.

• Dynamic parameters such as stroke volume variation, pulse pressure variation, and pleth variability index offer superior reliability in predicting fluid responsiveness compared to static parameters, reducing complications from unnecessary fluid administration.

• The integration of advanced hemodynamic parameters, including ventriculo-arterial coupling, arterial elastance, and cardiac power output, facilitates a more comprehensive understanding of cardiovascular dynamics, enabling individualized therapeutic strategies.

• Echocardiography serves as a cornerstone for hemodynamic assessment, offering valuable insights into preload, contractility, and valve function.

## Introduction

Advanced hemodynamic monitoring tools have revolutionized the field of perioperative medicine and critical care by providing detailed insights into the cardiovascular status of patients. These tools enable clinicians to assess and manage complex hemodynamic parameters such as cardiac output (CO), systemic vascular resistance, fluid responsiveness, and tissue perfusion with greater precision. The clinical significance of these monitoring techniques lies in their ability to detect subtle physiological changes that may indicate impending complications, allowing for timely interventions. By interpreting these advanced data, clinicians can tailor therapeutic strategies to optimize patient outcomes, particularly in critically ill patients and those undergoing major surgeries.

CO measurement has evolved significantly over time. In 1870, Adolf Eugen Fick used oxygen consumption and arterial-venous oxygen differences for the calculation of CO. The mid-20^th^ century introduced indicator dilution methods, including thermodilution with pulmonary artery catheters. The 1980s saw the rise of pulse contour analysis (e.g., PiCCO) and esophageal Doppler, enabling continuous monitoring; and, in the early 21^st^ century, lithium dilution (LiDCO) and bioimpedance/bioreactance (e.g., NICOM) emerged as less invasive options. By the 2010s, devices like MostCare Pressure Recording Analytical Method (PRAM) and advanced echocardiography provided real-time and non-invasive solutions.^1^ These innovations have continually enhanced the precision and practicality of CO measurement.

In Part I of this series, we examined the fundamental principles of hemodynamics, emphasizing that macrohemodynamic and microhemodynamic parameters are crucial for ensuring adequate tissue perfusion and guiding clinical decision-making in perioperative settings. Building on this foundation, Part II investigates advanced hemodynamic monitoring tools that have transformed perioperative medicine and critical care. These technologies provide more comprehensive insights into cardiovascular physiology, enabling clinicians to assess and manage complex parameters, such as CO, systemic vascular resistance, fluid responsiveness, and tissue perfusion, with enhanced precision. Table 1 organizes the various CO measurement techniques based on their underlying principles, devices, invasiveness, clinical applications, advantages, and limitations.

### Determinants of Blood Volume

Predicting fluid status and fluid responsiveness in critically ill patients is essential for identifying those who will benefit from volume expansion and more importantly, for avoiding fluid administration in patients who would not respond. Preload refers to the end-diastolic blood volume in the ventricles, reflecting the patient’s blood volume. The Frank-Starling law explains the relationship between preload and ventricular performance. In a healthy individual, located on the ascending portion of the Frank-Starling curve, fluid therapy increases stroke volume (SV) and consequently CO. However, as the myocardial fiber length continues to increase, tension and force begin to decrease beyond a certain point.^2^ Therefore, fluid replacement when the actual status is close to the “flat” part of the curve has little effect on CO and may result in overload, tissue edema, and ventricular dysfunction. The shape of the Frank-Starling curve depends on the patient’s cardiac contractility and afterload.^3^ In cases of systolic heart failure where left ventricular contractility is impaired, cardiac performance decreases for a given preload, shown by a downward shift of the normal curve. In contrast, an increase in left ventricular contractility due to vasopressor, and/or inotropic medication results in greater cardiac performance for a given preload, represented by an upward shift in the normal curve. Changes in afterload, the resistance the ventricle must overcome to initiate systole, will also shift the Frank-Starling curve. A reduction in afterload, similar to an increase in inotropy, causes the ventricular performance curve to shift upwards, while an increase in afterload shifts the curve downward, resembling a decrease in inotropy.

### Preload Assessment

Various pressure and volumetric parameters are used to assess preload. Central venous pressure (CVP) has been traditionally used for evaluating right ventricular preload and volume status. Transmural CVP (measured by subtracting end-expiratory intrathoracic pressure from the end-expiratory CVP) is assumed to reflect right ventricular filling pressure. Elevated transmural CVP may indicate right heart failure, necessitating echocardiographic evaluation for a definite diagnosis.^4^ Pulmonary artery occlusion pressure (PAOP), which reflects pressures in the pulmonary vasculature and left atrium, is an estimate of left ventricular end-diastolic pressure. Elevated PAOP may indicate severe left heart failure or significant mitral stenosis. Although both PAOP and CVP reflect the filling pressures of the left and right ventricles, they are static parameters with poor correlation to fluid responsiveness and have limited utility in goal-directed fluid therapy.^5^ Another pressure parameter, mean systemic filling pressure (Pmsf), refers to the “theoretical” pressure when blood is evenly distributed throughout the systemic circulation once the heart stops pumping. Pmsf values lie between the mean arterial pressure (MAP) and CVP, and Pmsf is more closely related to stressed blood volume. While Pmsf can be used to assess volume status accurately, it is difficult to measure and has limited clinical use.^6^ Volumetric parameters such as global end-diastolic volume (GEDV) and extravascular lung water (EVLW), provide more accurate assessments of ventricular preload when compared to filling pressures. GEDV, the volume of blood in all cardiac chambers at the end of diastole, especially correlates with cardiac preload and can assess whether preload has sufficiently increased during volume loading.^5^ Furthermore, it can be used even with non-sinus rhythms and spontaneous ventilation. EVLW refers to fluid accumulated in the extravascular space of the lungs, such as in the interstitial and alveolar spaces. It increases with higher hydrostatic pressures (fluid loading) or increased pulmonary vascular permeability [as in acute respiratory distress syndrome (ARDS)]. EVLW can help in diagnosing and assessing the severity of pulmonary edema and ARDS. However, like the other static variables, GEDV and EVLW are less reliable indicators of fluid responsiveness compared to dynamic variables.^6^

### Fluid Responsiveness

**Figure 1:**
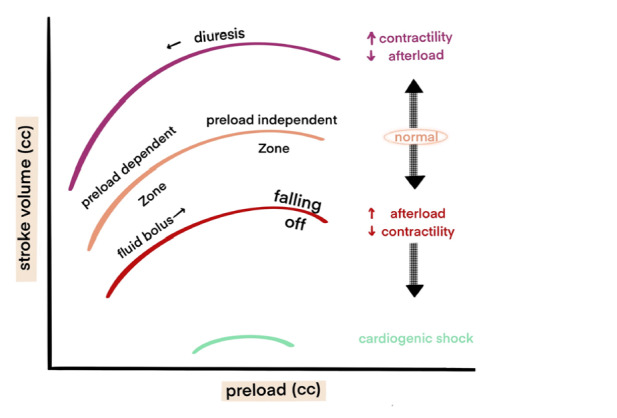
Frank-Starling curve.

Fluid responsiveness is traditionally defined as a 10-15% increase in SV or CO following a fluid bolus.^7^ In moderate- and high-risk patients or surgeries, inadequate intravenous fluid (IV) replacement may lead to insufficient tissue perfusion, cellular hypoxia, and subsequent organ dysfunction or failure.^8^ Conversely, poor outcomes are also associated with excessive fluid loading.^9, 10^ Positive fluid balance can cause interstitial edema, impaired microvascular flow, and an increase in CVP (limiting venous return). Studies across various patient populations show that only about 50% of hemodynamically unstable patients are fluid responders.^11, 12^ As such, the primary reason for fluid loading in patients is to increase CO. Therefore, reliable determinants of fluid responsiveness should be selected based on individual hemodynamic responses and applied only when an increase in CO is anticipated (Figure 1).

Previously, static parameters such as filling pressures and volumetric changes were used to predict fluid responsiveness, but studies have shown that these static indicators do not reliably do so.^5^ Instead, dynamic variables, such as SV variation (SVV), systolic pressure variation (SPV), and pulse pressure variation (PPV), which are measured using a less invasive method based on the interaction between the heart and lungs during mechanical ventilation, have been shown to predict fluid responsiveness more reliably.^13^ Additionally, several clinical studies using SVV and PPV to guide individualized fluid therapy have demonstrated reduced postoperative complications and shorter hospital stays.^14^ The pleth variability index (PVI), a non-invasive alternative to PPV, is also based on changes observed during the respiratory cycle. Other intermittent dynamic maneuvers, such as passive leg raising (PLR), fluid loading tests, and the end-expiratory occlusion test (EEOT), have proven useful in predicting which patients will benefit from volume expansion.^15^ Dynamic parameters have also shown potential benefits in reducing the duration of mechanical ventilation in patients with fluid overload.^16^ The use of these dynamic parameters in guiding fluid therapy has been widely accepted and recommended in guidelines.^17, 18^

During “positive pressure” ventilation, an increase in intrathoracic pressure during inspiration leads to decreased venous return (right ventricular preload) and SV. At the same time, the afterload on the right ventricle increases due to the rise in transpulmonary pressure during inspiration. The decrease in right ventricular SV subsequently leads to a reduction in left ventricular filling. This change is minimal during expiration.

**Figure 2:**
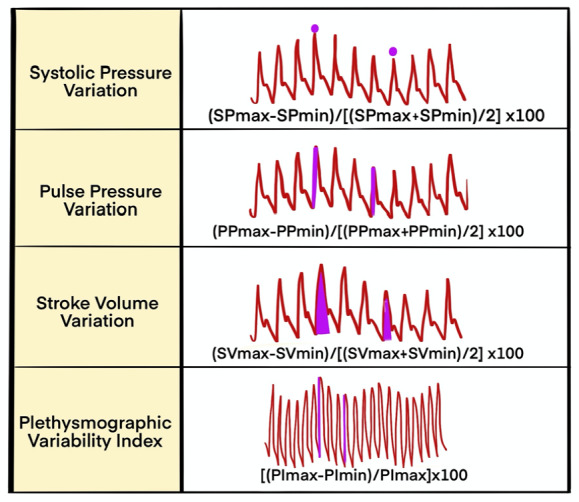
Illustration of dynamic hemodynamic parameters used for assessing fluid responsiveness in mechanically ventilated patients.

SVV and PPV are calculated as the ratio of the maximum difference in the values measured during a single ventilation cycle (Figure 2). SPV is the difference between the maximum and minimum systolic arterial pressures during a mechanical breath. Increased SV and PPV are more pronounced in fluid responder patients, as they lie on the steeper portion of the Starling curve (Figure 2).^19^ In patients receiving controlled mechanical ventilation with tidal volumes ≥7-8 mL kg^-1^ of ideal body weight (IBW), a PPV greater than 13% strongly suggests existing fluid responsiveness, while a PPV of less than 9% is unlikely to be fluid-responsive. Values between 9% and 13% refer to inconclusive results (the “gray zone”).^20, 21, 22^ PPV is generally considered the most accurate dynamic variable under mechanical ventilation and is often regarded as the gold standard for evaluating new dynamic parameters.^23^ Threshold values for SVV are also defined within the 9-13% range. Although SVV is slightly less accurate than PPV, this is likely due to the computational limitations of real-time pulse wave analysis (Figure 2).

Several limitations exist when interpreting PPV/SVV. Obtaining accurate results from these monitors requires good-quality arterial waveform data. On the other hand, PPV decreases at high respiratory rates (30-40 breaths per minute) regardless of fluid status.^24^ Patients should be mechanically ventilated with adequate tidal volumes, and without any spontaneous respiratory efforts. The hemodynamic effects of spontaneous respiration are different from those during mechanical ventilation. During spontaneous inspiration, intrathoracic pressure decreases, increasing venous return and SV. The effects of this pressure change can vary from breath to breath. Conversely, in a hypovolemic shock patient with spontaneous breathing, inspiration can cause vena caval collapse, leading to reduced venous return, and blood pressure (pulsus paradoxus). Thus, dynamic parameters represent limitations in patients with spontaneous breathing and may require additional maneuvers. For example, combining dynamic parameters with maneuvers that provoke cyclic changes of intrathoracic pressures (e.g., deep inspiration or forced inspiratory breathing) has been shown to improve the assessment of fluid responsiveness.^25^ Recently, PPV has been reported to predict fluid responsiveness in patients who are spontaneously breathing with mechanical assistance and generate low inspiratory effort (airway occlusion pressure <1.5 cmH_2_O).^26^ Another factor that limits the use of dynamic variables in clinical practice is the necessity of very low tidal volumes (e.g., 6 mL kg^-1^ IBW or less) for lung-protective ventilation modality, which is preferred in such conditions as ARDS. During low-tidal ventilation, low PPV does not rule out fluid “responsiveness”, yet high PPV still indicates the existence of such responsiveness.^27^ Considering those limitations, alternative maneuvers have been proposed. For instance, an absolute increase of PPV/SVV via a tidal volume challenge (a temporary increase from 6 mL kg-1 IBW, to 8 mL kg^-1^), or an absolute decrease via a mini-fluid challenge may help predict fluid responsiveness.^28, 29^ Contrary to what is normally expected, SVV is shown to be a stronger predictor of fluid responsiveness than PPV during lung-protective mechanical ventilation (<8 mL kg^-1^ tidal volume, no arrhythmias or spontaneous respiratory efforts), particularly in cardiac surgery or septic shock patients.^30^

Cardiac arrhythmias, severe aortic valve disease, and right and left ventricular failure are additional limitations of PPV and SVV. Several mechanisms are valid for this proposition. For example, arrhythmias cause increased variability in SV. In patients with right heart failure, mechanical ventilation-induced preload may reduce right ventricular CO, which may be mistakenly attributed to hypovolemia or pneumoperitoneum. Increased intra-abdominal pressure reduce thoracic compliance and may lead to incorrect interpretation of PPV/SVV. Reduced venous return due to increased intra-abdominal pressure leads to preload dependence, which causes a false-positive misinterpretation. Similar issues can occur in different surgical positions (prone, lateral etc.), where intra-abdominal pressure and lung compliance are affected, and dynamic parameters must be interpreted with caution. Increased lung and chest wall compliance, air trapping, and high tidal volumes, inflation pressures, or positive end-expiratory pressure (PEEP) settings may also cause false positives, where dynamic indices rise without true fluid responsiveness. Lastly, these methods are also inefficient for predicting fluid responsiveness under open-chest conditions during cardiac and/or thoracic surgery.

In cases where dynamic parameters are questionable, alternative tests such as the PLR test, EEOT, lung recruitment maneuvers, or mini-fluid tests are recommended. In particular, both tidal volume challenge and EEOT have been shown to be good predictors in critically ill patients ventilated with lower tidal volumes (<8 mL kg^-1^) without arrhythmias or respiratory effort.^30^ EEOT involves pausing the ventilation at the end of expiration for 15 seconds, which causes the airway pressure to drop to the actual positive end-expiratory pressure (PEEP) level, thus reducing intrathoracic pressure and increasing cardiac preload. The diagnostic threshold for this test is a >5% increase in CO.^31^ In a different clinical scenario, elevated PPV and SVV values accompanied by hypotension following anaesthesia induction may result from widespread vasodilation; in such situations, vasoactive treatment can be considered, and whether or not fluid bolus therapy is administered. Similarly, in sepsis or cardiac surgery patients, dynamic variables may be elevated with vasoplegia itself. While vasopressors may increase preload, inotropes shift the Starling curve upwards, potentially altering the clinical assessment of true volume deficit. Therefore, in situations with reduced CO, fluid responsiveness should be evaluated along with afterload and contractility parameters in a comprehensive manner.

Non-invasive methods for fluid responsiveness assessment include pulse oximeter plethysmographic waveform amplitude changes (ΔPOP) and the automatic PVI.^32^ The perfusion index (PI) is the ratio of pulsatile to non-pulsatile blood flow in the capillary bed. The PVI is calculated as the ratio of the difference between the maximum and minimum PI values to the maximum PI, representing the ratio of pulsatile to non-pulsatile infrared light absorption. PVI, an indirect marker of PPV, predicts fluid responsiveness with a cut-off of 14% based on an algorithm that continuously evaluates ΔPOP using the PI, where mechanical ventilation is required.^33, 34^ While the limitations of invasive dynamic parameters also apply to PVI, its reliability is further reduced in cases of hypothermia, circulatory failure, vasoactive drug use, and vasoconstriction.

An alternative dynamic test for assessing fluid responsiveness is the fluid loading test. Since the ideal organ perfusion value for a patient is unknown, fluid titration using small incremental boluses (100 to 250 mL crystalloid infusion over 5 to 10 minutes limited to 4 mL kg^-1^) guided by changes in SV, and PP is recommended.^35^ More than 10-12% increase in SV, observed one minute after fluid infusion, indicates fluid-responsiveness, exhibiting blood flow and tissue perfusion recovery via the replacement. The absence of an increase following fluid bolus is the most reliable indicator of potential congestion and edema due to additional volume. The “mini-fluid” loading test involves the rapid infusion of 100 mL IV fluid, and is assumed to predict an increase in SV as though 500 mL had been infused. However, excessive or repeated volume boluses may lead to undesirable fluid overload and reduced oxygen delivery due to hemodilution in patients who are not fluid-responsive.^36^

Another method to assess fluid responsiveness is the PLR test. The patient is positioned with their legs raised at a 45-degree angle while the upper body remains flat, resulting in a semi-recumbent position. This maneuver helps return approximately 300 mL of venous blood from the abdomen and lower extremities to the heart. A more than 10% increase in CO after PLR has been shown to reliably predict fluid responsiveness in adults with acute circulatory failure.^37^ PLR-induced changes in CO reliably predict fluid responsiveness; however, arterial blood pressure is often monitored instead of CO. In such cases, it is important to note that PLR-induced changes in PP or SAP can serve as a feasible alternative, albeit with lower predictive accuracy.^38^ This finding highlights that relying solely on blood pressure changes during PLR can be misleading, and the use of CO monitoring is recommended. PLR is a reversible, repeatable, and easy-to-perform test. In situations such as spontaneous respiration, cardiac arrhythmias, low tidal volume ventilation, and low lung compliance, PLR may represent advantages over SVV-based indices.^31^ Performing the test may be difficult or directly interfere with ongoing surgical procedures and requires special attention. On the other hand, clinical limitations of PLR may include amputated lower extremities, severe hypovolemia, and intra-abdominal hypertension. Other limitations include potential increases in intracranial pressure, reduced lung compliance, and increased aspiration risk. It is important to note that, unlike the mainstream dynamic parameters, those alternative dynamic tests (fluid loading, EEOT, and PLR) are measured “intermittently”. Continuous dynamic variables can detect hemodynamic changes much earlier than intermittent variables, offering significant clinical benefits.

### Ventriculo-Arterial Coupling

Ventriculo-arterial coupling (VAC) is a macrohemodynamic parameter that reflects the mechanical interactions between the ventricle and the arterial system. Although the left ventricle and aorta have distinct volume dynamics, they converge at a single common point defined as end-systolic pressure (ESP). At this pressure, effective arterial elastance (Ea) is calculated based on the SV ejected into the arterial system, while ventricular elastance (Ees) is derived from the remaining volume within the ventricle. ESP should ideally be measured at the dicrotic notch pressure; however, when this is not feasible, it can be approximately calculated as 90% of the systolic blood pressure, since the aortic valve closes shortly after the systolic descent. The Ea/Ees ratio, a dimensionless parameter, defines VAC and quantifies the coupling between the arterial system and the ventricle. VAC serves as a critical determinant of myocardial oxygen demand and ventricular contraction efficiency. In the cardiovascular system, energy balance is optimal when VAC equals 1 (normal values: 1.0±0.36), while maximum cardiac efficiency is observed at a VAC ratio of approximately 0.5.^39^ Deviations in this ratio are not only indicative of disease severity but also serve as an independent predictor of clinical outcome. VAC represents a measure of cardiac work efficiency. A thorough understanding of arterial elastance (a parameter of afterload) and Ees (a parameter of contractility) is essential for interpreting and utilizing VAC effectively.

### Afterload, Arterial Impedance, and Arterial Elastance

Afterload is defined as the total load or resistance the ventricle encounters while pumping blood into the arterial system. Although this qualitative definition is partially accurate, a quantitative description is only possible through the concept of arterial impedance, which reflects the resistance blood faces while traversing the arterial system. This resistance depends on the physical properties of arteries (e.g., elasticity and diameter) and the characteristics of blood flow (e.g., viscosity). While vascular resistance applies to steady flows, pulsatile flows can only be described using impedance.^40^ Arterial impedance is typically calculated via frequency analysis of blood pressure and flow. In this analysis, harmonics (repeating sinusoidal waves) at different frequencies are considered.^41^ The ratio of a pressure harmonic to a flow harmonic at the same frequency is termed impedance at that frequency. The structural characteristics of arteries (e.g., size and elasticity) determine the characteristic impedance, which represents the pressure-to-flow ratio as a wave propagates along the artery.^41^ However, the arterial system also includes reflected pressure and flow waves originating distally. “Input impedance” is the term for combined effects of all arterial components, including characteristic impedance and reflected waves, and is thus a more comprehensive measure of afterload.

PRAM is used for CO monitoring via pulse contour analysis, that incorporates arterial impedance (Ztot = mmHg·s mL) for SV measurement. While ztot shows a strong clinical correlation with Ea (mmHg mL^-1^), the inclusion of time (s) as a factor distinguishes ztot from Ea, offering a more dynamic perspective by taking the temporal effects on pressure into account. Although normal ztot ranges have not been definitively established in the literature, the authors of this manuscript propose an ideal range of 0.35-0.8 mmHg·s mL based on their experience when accurate arterial waveforms and stable SVs are observed. Additionally, the correlation between ztot and Ea is crucial for accurate and reliable CO data. Though conditions like arterial vasospasm may lead to discordance between these parameters, highlighting a potential limitation of these methods.

Ea is a parameter encompassing peripheral resistance, vascular compliance, characteristic impedance, and systolic-diastolic time intervals. Ea can also be calculated as the ratio of ESP to SV (ESP/SV) derived from the pressure-volume loop. A more than 90% correlation between Ea and input impedance has been described.^42^ Ea offers a simplified yet highly useful approach for assessing afterload in clinical practice.

### Ventricular Elastance

Although myocardial contractility is a primary determinant of ventricular systolic function, ventricular performance is not solely limited to contractility. Structural properties of ventricular myocytes (e.g., elastance, compliance, fibrosis), synchronized involvement in contraction, and geometric remodeling of the cavity significantly influence performance as well.^43^ In clinical practice, the most common method for assessing ventricular systolic function is the estimation of ejection fraction (EF) via echocardiography. EF is an important indicator in heart failure and acute myocardial infarction; it is accepted as a major factor in therapeutic decision-making. However, EF is a preload-dependent and afterload-sensitive parameter. On the other hand, Ees is a measure derived from the complex interplay between inotropic activity and the structural, geometric, and functional properties of the Ees is recognized as a true intrinsic indicator of ventricular inotropy.^44^ It is calculated by dividing ESP by ventricular end-systolic volume (ESP/ESV).^45^ The modified single-beat method, as described by Chen et al.,^46^ is the only validated non-invasive approach for measuring Ees when compared to invasive gold-standard methods. This technique calculates Ees in a single cardiac cycle by integrating systolic and diastolic blood pressures with echocardiographic parameters, including ventricular end-systolic and end-diastolic areas, EF, SV, pre-ejection period, and systolic time interval.

Advanced hemodynamic monitoring allows easy interpretation of VAC through the relationship between Ea and Ees, offering insights into varying hemodynamic states. While Ees is preferred in VAC calculations due to its unique role as an intrinsic predictor of contractility, its subjective reliance on echocardiographic measurements remains a limitation. Alternative parameters for assessing contractility should also be considered.

### dp/dt and dp/dt(max) as Measures of Barometry

The dp/dt, the first derivative of the rise in left ventricular pressure, and dp/dt(max), representing its maximum rate of increase, are parameters used to assess myocardial contractility. These values can also be calculated non-invasively using echocardiography by measuring the slope of the regurgitant jet velocity from the mitral valve to the left atrium over time. However, ventricular dp/dt(max) measurements are often invasive, technically demanding, and or dependent on the presence of mitral regurgitation, limiting their routine application.

In contrast, arterial dp/dt(max) can be evaluated through pulse contour analysis by measuring the slope of the upstroke in the arterial waveform’s anacrotic limb over time. Normal values are between 0.9-1.3 mmHg msec; excessively high values (e.g., >1.7 mmHg msec) may indicate underdamping in arterial waveform analysis, as they may not correlate with physiology.^47, 48^

Correlation among dp/dt(max) measurements obtained from various anatomical sites under differing hemodynamic conditions has been demonstrated.^47^ However, arterial dp/dt(max) measurements can be inaccurately low in conditions such as insufficient preload, low afterload, or arrhythmias affecting ventricular filling time.^48^ Additionally, in cases of aortic stenosis or dynamic left ventricular outflow tract obstruction, the correlation between left ventricular and arterial measurements may be disrupted.

### Cardiac Function Index and Global Ejection Fraction

Cardiac function index (CFI) and global ejection fraction (GEF) are easily measured parameters with the PiCCO system. CFI equals the ratio of CO to GEDV (CO/GEDV), while GEF equals the ratio of SV to GEDV/4. Due to having a temporal component, CFI is considered a more dynamic parameter. In contrast, GEF, which lacks a time constant, is accepted as a static parameter and is recommended for trend monitoring to correlate with preoperative EF. In patients with right ventricular dysfunction, these measurements may be misleading, and echocardiographic evaluation of right ventricular involvement is advised. Despite being preload-dependent, both CFI and GEF are reliable indicators for assessing cardiac contractility.^49^

### Cardiac Power Output

Cardiac power output** (**CPO) measures the heart’s pumping capacity by evaluating the work performed through pressure and volume. It is calculated using the formula CPO=COxMAP/451 and is expressed in watts. This parameter reflects the myocardial contractility reserve required to maintain the current hemodynamic state, based on two key parameters targeted in hemodynamic management. In conditions with low cardiac performance, such as heart failure, a low CPO value is associated with poor prognosis. Additionally, CPO may be used for monitoring the response to treatment in cardiac diseases.^50^

### Cardiac Cycle Efficiency

The evaluation of the cardiovascular system involves various hemodynamic parameters, each providing insights into different aspects of its function. While these parameters offer specific measurements, it is valuable to express the system’s overall efficiency and individual patient conditions with a single variable. In this context, energy-based assessments have gained prominence. Cardiac cycle efficiency (CCE) emerges as a dimensionless parameter that evaluates the system’s global performance based on energy utilization. CCE is calculated as the ratio of energy expended during systole to the total energy expended throughout the cardiac cycle. Theoretically, a perfectly efficient system would have a CCE value of 1. However, due to entropy and other energy losses inherent in biological systems, this level of efficiency is never achieved. Under normal circumstances, CCE values are expected to range between 0 and 1. Negative values, however, indicate that the cardiovascular system is undergoing a hemodynamic compensation process. The clinical significance of CCE, as a global indicator of cardiovascular performance, underscores its utility in both comprehensive system evaluation and the development of personalized assessment and treatment strategies.^51^

### Dynamic Arterial Elastance (Eadyn)

Dynamic arterial elastance (Eadyn) is calculated as the ratio of PPV to SVV during the respiratory cycle. It is intended to reflect the responsiveness of the arterial system to pressure changes, thereby representing dynamic arterial tone characteristics. Unlike static indices of afterload, Eadyn offers real-time assessment and has been proposed as a tool for predicting MAP changes following fluid administration or the development of hypotension after the reduction of vasopressor support. Studies conducted under controlled mechanical ventilation have demonstrated that Eadyn can predict a MAP decrease greater than 10%, achieving a sensitivity of 71% and specificity of 89% at a cut-off value of 0.84 (area under the curve: 0.84).^52^ These findings suggest that Eadyn may serve as a valuable guide not only for fluid responsiveness but also for vasopressor titration. The use of Eadyn as a dynamic indicator of VAC can be better understood by considering the physiological relevance of its components. During the respiratory cycle, variations in PPV—under constant SV—are considered to reflect the elastic properties of the arterial system (Ea). Conversely, SVV represents the response of the left ventricle to fluctuations in preload and can be viewed as a dynamic surrogate of left ventricular contractility (end-systolic elastance, Ees). Accordingly, Eadyn may be interpreted as a continuously monitorable, non-invasive surrogate for the Ea/Ees ratio, reflecting VAC. Experimental studies have demonstrated a positive correlation between Eadyn and left ventricular mechanical efficiency, and a negative correlation between Eadyn and the Ea/Ees ratio.^53^ These data indicate that Eadyn is not merely a measure of variation, but a functional parameter that evaluates the interaction between CO and arterial load. Moreover, it has been reported that invasively measured Eadyn values during deep inspiration may play a significant role in predicting post-induction hypotension.^54^

### Role of Echocardiography in Hemodynamic Assessment

**Figure 3:**
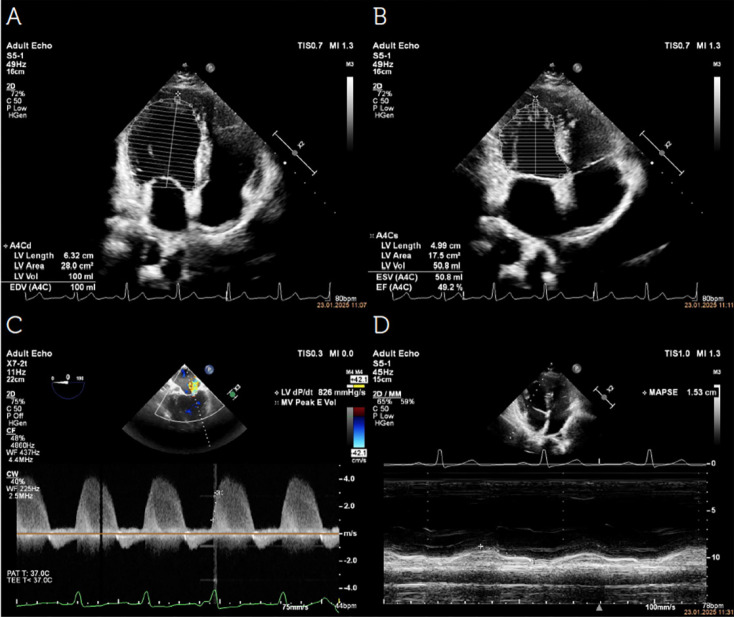
Echocardiographic assessment of left ventricular (LV) function. A) Apical four-chamber view demonstrating the LV end-diastolic volume (EDV), measured to assess LV filling and chamber dimensions. B) Apical four-chamber view illustrating the LV end-systolic volume (ESV), utilized for ejection fraction (EF) calculation. EF (%)=(LVEDV-LVESV)/(LVEDV)x100. C) Continuous-wave Doppler echocardiographic recording of mitral regurgitation (MR) jet, used to calculate dp/dt max, an index of LV contractility. The dp/dt max is determined by measuring the rate of pressure rise across the mitral valve during early systole. D) M-mode echocardiographic assessment of mitral annular plane systolic excursion (MAPSE), a parameter reflecting longitudinal LV function. MAPSE is obtained by tracking the systolic displacement of the mitral annulus, serving as a surrogate marker for global LV systolic performance.

**Figure 4:**
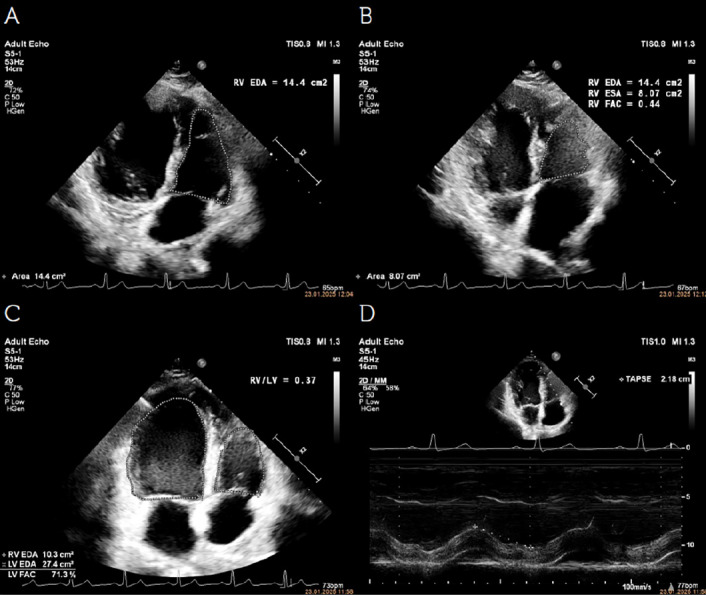
Echocardiographic assessment of left ventricular (LV) function. A) Apical four-chamber view demonstrating the LV end-diastolic volume (EDV), measured to assess LV filling and chamber dimensions. B) Apical four-chamber view illustrating the LV end-systolic volume (ESV), utilized for ejection fraction (EF) calculation. EF (%)=(LVEDV-LVESV)/(LVEDV)x100. C) Continuous-wave Doppler echocardiographic recording of mitral regurgitation (MR) jet, used to calculate dp/dt max, an index of LV contractility. The dp/dt max is determined by measuring the rate of pressure rise across the mitral valve during early systole. D) M-mode echocardiographic assessment of mitral annular plane systolic excursion (MAPSE), a parameter reflecting longitudinal LV function. MAPSE is obtained by tracking the systolic displacement of the mitral annulus, serving as a surrogate marker for global LV systolic performance.

**Figure 5:**
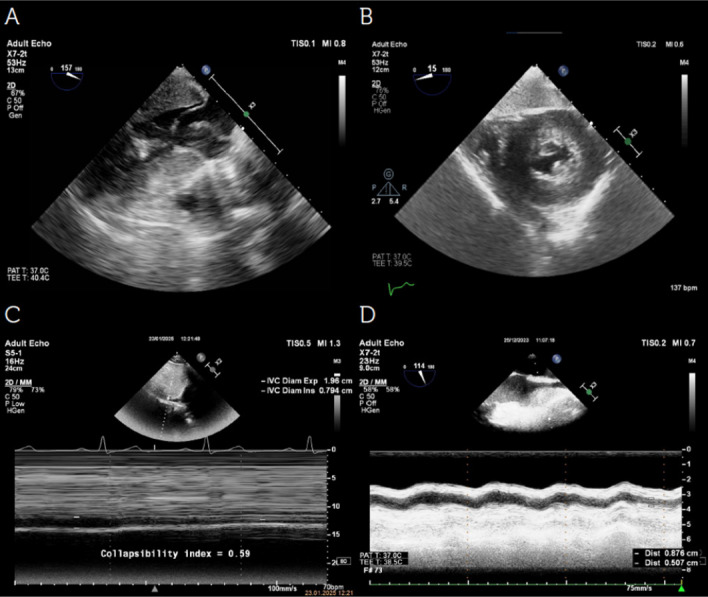
Echocardiographic assessment of volume status. A, B) Echocardiographic images demonstrating “kissing ventricles,” a hallmark of hypovolemia. In severe hypovolemia, left ventricular walls nearly touch at end-systole due to reduced preload, with minimal residual cavity. Additionally, diastolic filling is significantly impaired, further supporting volume depletion. C) Inferior vena cava (IVC) collapsibility index (IVC-CI) measurement using M-mode echocardiography in a spontaneously breathing patient. IVC-CI=(IVCmax–IVCmin)/(IVCmax)x100. >50% suggests a low central venous pressure (< 5 mmHg), indicating hypovolemia. IVC distensibility index (IVC-DI) is applied in mechanically ventilated patients and is calculated as follows: IVC-DI=(IVCmax–IVCmin)/(IVCmin)x100. >18% suggests fluid responsiveness. D) Superior vena cava (SVC) collapsibility index (SVC-CI) measurement using transesophageal echocardiography, which is primarily applied in mechanically ventilated patients. SVC-CI=(SVCmax-SVCmin)/(SVCmax)x100. >36-40% suggests volume responsiveness.

As a rapid diagnostic method, hemodynamic-focused echocardiography offers an excellent opportunity to examine signs of filling abnormalities, cardiac preload, myocardial contractility, and valve function (Figures 3, 4, 5). A summary of all echocardiographic findings, including clinical symptoms, provides a comprehensive evaluation of the patient’s cardiovascular function, thereby helping guide pathophysiology-focused and individualized hemodynamic treatment to optimize/maintain CO. Proper patient selection, acquisition, interpretation, synthesis, and the subsequent application of evidence-based therapy will continue to be a critical goal for future research, aiming to enhance the use of echocardiography in critically ill patients.

## Conclusion

In this review, we have examined advanced hemodynamic parameters and their integration into clinical practice, aiming to refine perioperative hemodynamic assessment with a more comprehensive and targeted approach. Accurate interpretation of key parameters such as CO, vascular resistance, fluid responsiveness, VAC, arterial elastance, and impedance is essential for maintaining hemodynamic stability in surgical patients. Furthermore, echocardiographic parameters provide valuable insights into myocardial function and volume status, contributing to a more nuanced understanding of perioperative cardiovascular dynamics. The transition from traditional static measurements to dynamic parameters enables more precise fluid and vasopressor management, ultimately optimizing perioperative patient care. The implementation of advanced hemodynamic assessment algorithms has the potential to reduce perioperative complications and enhance patient safety. Further integrating these parameters into clinical decision-making frameworks will further refine individualized patient management strategies, fostering advancements in perioperative hemodynamic optimization.

## Figures and Tables

**Table 1. Comparison of Cardiac Output Measurement Techniques:** 

**Classification**	**Principle**	**Devices**	**Advantages**	**Limitations**
Thermodilution-based monitors	Involves injecting a bolus of cold fluid into the venous circulation. The downstream temperature change is recorded, allowing cardiac output calculation	PAC, PiCCO	Direct measurement, high accuracy in unstable patients	Invasive Requires initial calibration Intermittent measurements Risk of infection and arrhythmias
Arterial waveform analysis	Analyzes the arterial pressure waveform to estimate stroke volume and cardiac output. The shape and area under the pressure curve are assessed, often calibrated by initial thermodilution or lithium dilution measurement	ProAQT Pulsioflex LiDCOrapid PRAM (Mostcare) (no external calibration) FloTrac/Vigileo	Continuous, minimally invasive	Requires dedicated transducer or initial calibration Affected by vascular tone and arrhythmias
Doppler ultrasound-based monitors	Measures blood flow velocity in major arteries, deriving stroke volume from velocity data combined with vessel diameter measurements	ED, TTE, TEE	Rapid ED can continue with arterial waveform analysis TTE non-invasive TEE, ED minimally invasive	Operator-dependent, intermittent measurements ED requires initial calibration
Bioimpedance and bioreactance-based monitors	Measures changes in thoracic electrical impedance or phase shifts (reactance) as blood flows through the aorta, calculating stroke volume from variations in impedance during the cardiac cycle	EC, Starling SV (bioreactance), NICaS (Bioimpedance)	Non-invasive, continuous	Requires external calibration sensitive to motion artifacts, electrocautery, and thoracic conditions, difficult use during intraoperative period

PAC, pulmonary artery catheter; ED, esophageal Doppler; TTE, transthoracic echocardiography; TEE, transesophageal echocardiography; EC, electrical cardiometry
